# Supplementary material for: Characterization of the Role of SPL9 in Drought Stress Tolerance in Medicago sativa
Source: Int J Mol Sci. 2020 Aug 20;21(17):6003. doi: 10.3390/ijms21176003 (PMC7504591; doi:10.3390/ijms21176003)
Supplement: Supplementary file 1 [file ijms-21-06003-s001.zip › Supplementary Files/Table S1.pdf]

Table S1. Primer specifications and how they were utilized in relation to this study.

Primer Name	Primer Sequence	Primer Use
SPL9RNAi_F	CACCCTCTCTCTTCTGTCAAATCAAACATGGG	SPL9-RNAi construct cloning
SPL9RNAi_R	TTACAGTGACCATTGAGAAGATTCATAGG	SPL9-RNAi construct cloning
nptII-F	GAGGCTATTCGGCTATGACTG	Forward primer for *SPL9*-RNAi transgene confirmation
nptII-R	ATCGGGAGCGGCGATACCGTA	Reverse primer for *SPL9*-RNAi transgene confirmation
LA-MsSPL9-Fq1	AGATACAGCTCTTGCTACTG	Forward primer of *MsSPL9* for qPCR
LA-MsSPL9-Rq1	GTTGAGAATGTTGAACTGAC	Reverse primer of *MsSPL9* for qPCR
DFR-F	GTTTGTGTCACAGGGGCTTC	Forward primer of *MtDFR* for qPCR
DFR-R	TTCAAGTTTTCTGGGTCGCG	Reverse primer of *MtDFR* for qPCR
Cat1-F	GAAAAGCTTGCCCAATTTGA	Forward primer of *MtCAT1* for qPCR
Cat1-R	ATGACGGGTGTCTGAACTCC	Reverse primer of *MtCAT1* for qPCR
GSH-F	ACGCTTCCCAGCTTTAATGA	Forward primer of *MtGSH* for qPCR
GSH-R	CCCCAACAAGAAGACCATTG	Reverse primer of *MtGSH* for qPCR
ms_ADF1qF	TCAAGGCGAAAAGGACACAC	Forward primer of *MsADF* for qPCR (reference gene)
ms_ADF1qR	AAAACAGCATAGCGGCACTC	Reverse primer of *MsADF* for qPCR (reference gene)
ms_eIF4AqF	TGCTAAGTTGCCTGAAACCG	Forward primer of *MseIF4A* for qPCR (reference gene)
ms_eIF4AqR	TGCCCATGTTTTCACCTTGC	Reverse primer of *Mself4A* for qPCR (reference gene)
