# Supplementary material for: Characterization of the Role of SPL9 in Drought Stress Tolerance in Medicago sativa
Source: Int J Mol Sci. 2020 Aug 20;21(17):6003. doi: 10.3390/ijms21176003 (PMC7504591; doi:10.3390/ijms21176003)
Supplement: Supplementary file 1 [file ijms-21-06003-s001.zip › Supplementary Files/Figure S1.pdf]

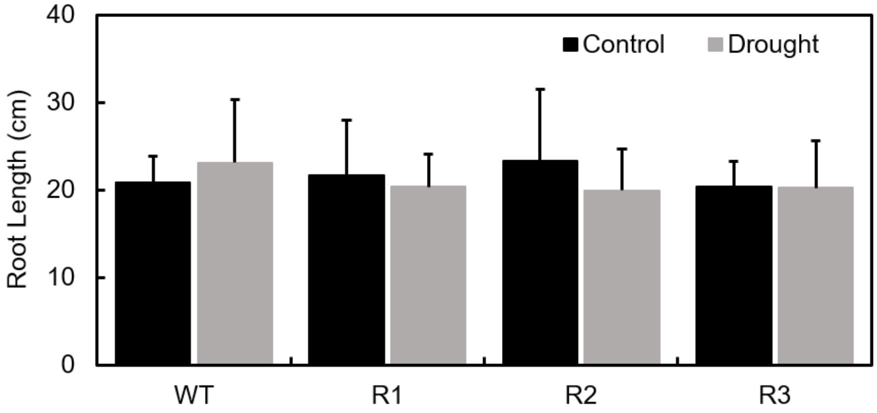

Figure S1: *SPL9*-RNAi root length in response to drought. Root length of plants grown in containers after 12 days of control and drought conditions. Asterisks indicate significant differences between treatments (*P*<0.05, where n=9-10, Two-Way ANOVA, Sidak test).
