# Supplementary material for: Characterization of the Role of SPL9 in Drought Stress Tolerance in Medicago sativa
Source: Int J Mol Sci. 2020 Aug 20;21(17):6003. doi: 10.3390/ijms21176003 (PMC7504591; doi:10.3390/ijms21176003)
Supplement: Supplementary file 1 [file ijms-21-06003-s001.zip › Supplementary Files/Figure S2.pdf]

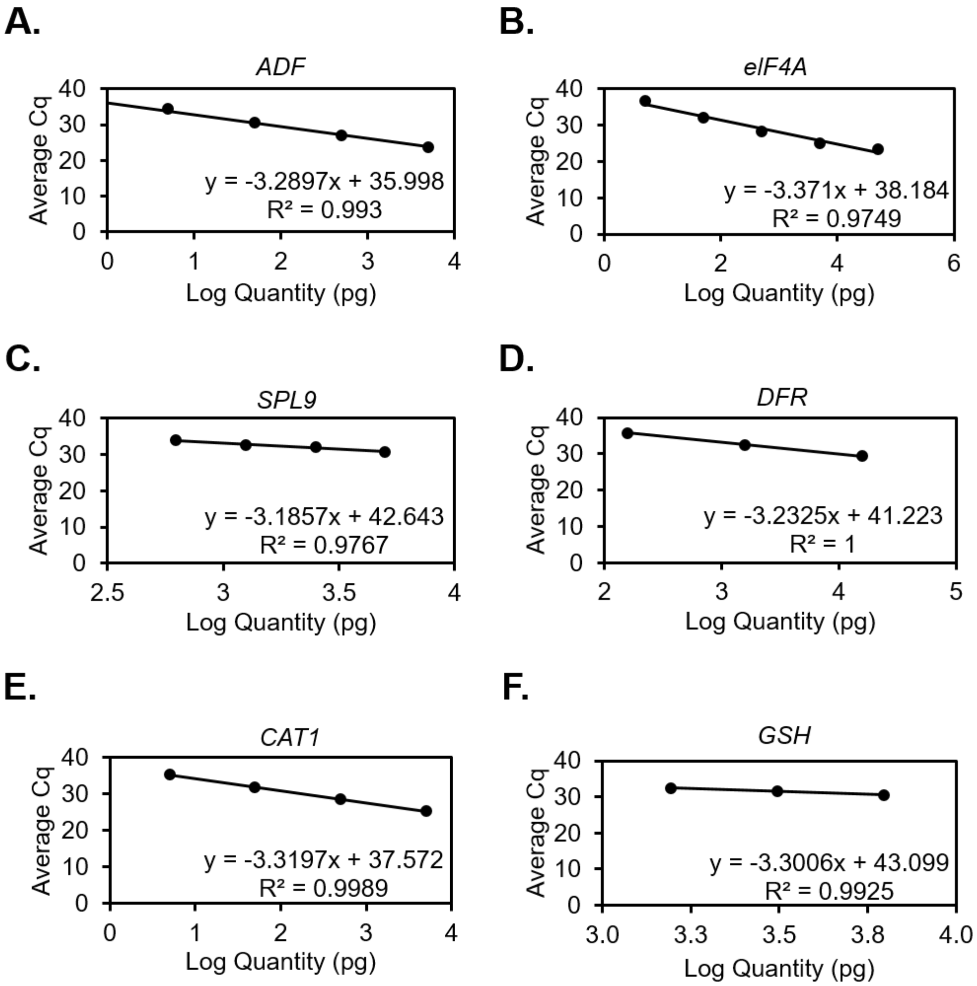

Figure S2: qRT-PCR standard curves. Standard curves for each primer pair used in qRT-PCR. Resulting equations were used to determine dynamic range and efficiency values.
