# Supplementary material for: Characterization of the Role of SPL9 in Drought Stress Tolerance in Medicago sativa
Source: Int J Mol Sci. 2020 Aug 20;21(17):6003. doi: 10.3390/ijms21176003 (PMC7504591; doi:10.3390/ijms21176003)
Supplement: Supplementary file 1 [file ijms-21-06003-s001.zip › Supplementary Files/Figure S3.pdf]

ATACGCTGCACTGCATCACTGCACTGAACTGGGTTCCTGCCAACAATACATAT
GGCCCAGCAGTGTCAACTCACTTCTTCCTTCCACCTCTTCCTCTTTTGTCTTC
CACTTCACTTTTCCACTATCATTCACTCTCTTCTTTTTTTAATGTTTTCACTT
TAACCAACAATAACAACCACCACTCCCCAGACCCCCCTCACCTATACTATTCT
CTCACTCTTACACTCACACTTCACTTCACTTCACTTATCTCTCACCAATGAAC
ACAATCTAGCAACCACCACCAAACCTCACACCAATGGATTCAGGAGGCAACTC
TTCTTCGGAAGAGTCCTCTCTTAATGGCTTAAAATTTGGCCAACGAATCTATT
TTGAAGATACAGCTCTTGCTACTGCTGCTGCTGCTACTTCTACCACCATTGCT
GCTAGTTCTTCTTCTTCTTCTGGTTCAAAGAAAGGAAGAGGTGGGTCAGTTCA
ACATTCTCAACCACCTCGGTGTCAAGTTGAAGGATGTAAACTAGATCTGACTG
ATGCTAAAGCTTACTATTCTAGACACAAAGTTTGTAGCATGCACTCTAAGTGC
CCAACTGTTACTGTTTCTGGTCTACAACAAAGGTTTTGTCAACAATGTAGCAG
ATTTCATCAGCTTGCTGAGTTTGATCAAGGAAAAAGAAGTTGCCGGAGACGAC
TAGCTGGTCATAACGAGCGTCGCAGAAAGCCCCCACCCAGCTCTCTCTTAACC
TCACGTTTTGCCAGGCTTTCTTCATCTGTTTTTGGTAACAGTGACAGAGGTGG
CAGCTTCTTGATGGAATTTGCTTCAAACCCAAAACTTAGTCTGAGAAATTCAC
TTCCACCACCCGGAAATCAGACCACAACAATCGGTTGGCCTTGGCCGGGGAAC
ACGGAGTCGCCATCTGACAACCTTTTCTTGCAAGGTTCGGTGGGTGGGACAAG
CTTCCCTGGTGCCAGGCATCCTCCCGAGGAAACTTACACCGGAGTCACAGATT
CAAACTGTGCT**CTCTCTCTTCTGTCAAATCAAACATGGG**GTTCTCGAAACACA
GAACCAAGTCCTGAATTGAATAACATGCTGAATTTCAATGGGACATCCATGAC
ACAACATGCTACATCTTCTCATGGTGTAGCCATGCATCAAATTCCAAACAATT
ACGAGGTTGTCCCTGATCTTGGTCGGGGTCACATTTCGCAGCCTCTTGGTAGC
CAACTCTCTGGTGAGCTTGATCTGTCGCAGCAGGGAAGGAGGCATTATATGGA
TGTAGAACATTCCAGGG**CCTATGAATCTTCTCAATGGTCACTGTAA**TGCACTT
GTTTGCTTTCAGGTTTGTAATAACATGTTTCACAAATATTTGAACTCAGGAAA
GTGAGAAGTGAACTAAGGCATACTTGATGCTCTTGCTTGTTTTGGTTTGTTTA
AACTGTTAGGCAAGGTGGGGCTAGCCTTGCTTCACTTTGTGGTTTGTAATCTC
TTCCTAGTTATTTGAGATTATCATGGTTTCAAATTTCAGGAAGTTGTTTGATG
TGGATTTGGTTGCACCTTTGTAGCATTGTGATTGTGAAAATTGCAAATAAATG
TTGCAATAGCGGCTTGAATCAATTTTATGTTTGCATTGAATGAT

Figure S3: *SPL9*-RNAi construct target location in *MsSPL9* coding sequence. Full length *Medicago sativa SPL9* cDNA [28] used as reference in the design of the *SPL9*-RNAi construct. To target *MsSPL9,* the 300 bp fragment (primers bolded) used in the RNAi construct was designed within the protein coding sequence (highlighted in yellow) but outside the conserved SBP domain (underlined). The region complimentary to miR156 is shown with red text.
